# A Physiology-Based Mathematical Model to Understand Drug Delivery from Contact Lenses to the Back of the Eye

**DOI:** 10.1007/s11095-023-03560-7

**Published:** 2023-07-27

**Authors:** Nadia Toffoletto, Benilde Saramago, Ana Paula Serro, Anuj Chauhan

**Affiliations:** 1 Centro de Química Estrutural, Institute of Molecular Sciences, Instituto Superior Técnico, Universidade de Lisboa, Av. Rovisco Pais, 1049-001 Lisbon, Portugal; 2 Centro de Investigação Interdisciplinar Egas Moniz, Instituto Universitário Egas Moniz, Quinta da Granja, Monte de Caparica, 2829-511 Caparica, Portugal; 3 Chemical and Biological Engineering Department, Colorado School of Mines, Golden, CO 80401 USA

**Keywords:** back of the eye, contact lens, drug delivery, mathematical model, pharmacokinetics

## Abstract

**Objective:**

Therapeutic contact lenses, able to store drug and deliver it to the eye surface in a sustained fashion, gained interest as an effective and patient-friendly alternative to eye drops. Recent animal studies also demonstrated the presence of therapeutic drug levels in the back of the eye after wearing drug-loaded contact lenses, thus opening the possibility of treating the posterior segment without need of invasive intraocular injections. The drug pathways from contact lenses to the back of the eye require further investigation.

**Methods:**

A mechanistic mathematical model was developed to evaluate the drug concentration over time in the tears, sclera and choroid, retina, aqueous humor and vitreous humor after the application of a therapeutic contact lens. The main drug transport mechanisms of the eye and the barrier properties of the different tissues were included in the model. Validation was performed by comparison with experimental data in literature.

**Results:**

The model predictions of drug concentration over time reflected the experimental data both in the anterior and posterior segment of the eye. The model can differentiate between contributions to transport from different pathways.

**Conclusions:**

The model constitutes a first step towards the possibility of predicting the ocular drug distribution and the treatment efficacy in the early stage of contact lens development, and it may help reduce both the need for *in vivo* tests (with ethical and economic advantages) and the gap between the lens design and clinical application. It also allows for an improved understanding of drug transport in the eye.

**Graphical abstract:**

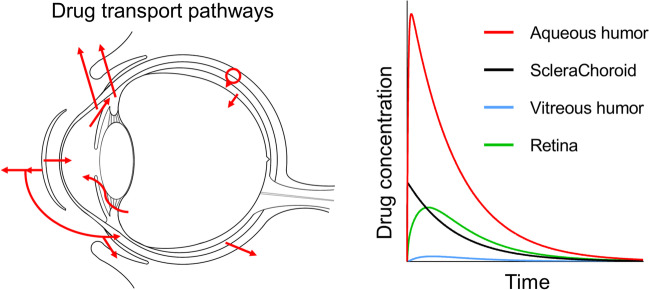

**Supplementary Information:**

The online version contains supplementary material available at 10.1007/s11095-023-03560-7.

## Introduction

Due to the ageing of population, chronic pathologies affecting the posterior segment of the eye, such as macular degeneration, diabetic retinopathy and macular edema, are affecting a growing number of patients worldwide [1]. The standard treatment for these conditions is performed by intravitreal injections, which allows overcoming the anatomical ocular barriers and delivering high drug doses in the posterior segment. However, such injections are invasive for the patient, usually require a monthly administration and are associated with side effects [2]. The use of injectable drug-eluting intravitreal implants, able to provide a therapeutic drug concentration for several months [3], reduces the frequency of injections but does not overcome the risk of adverse events associated with them.

The possibility of using soft contact lenses (SCLs) for ocular drug delivery gained interest as a patient-friendly approach involving a non-invasive and easily replaceable device, which, if compared to eye drops, can provide a sustained delivery over time and a longer drug residence time on the cornea, thus increasing the therapeutic efficiency. While most of the current research on drug-eluting SCLs focuses on the treatment of pathologies of the anterior segment [4–7], animal tests recently evidenced the presence of therapeutic drug levels also in the vitreous, choroid and retina after SCLs wearing [8–11]. These findings imply the presence of drug transport pathways from the anterior to the posterior segment, which would allow for the use of non-invasive topical drug delivery systems for the treatment of the back of the eye.

Understanding the ocular physiology and pharmacokinetics is fundamental for the design of effective therapies and devices. Mathematical models constitute an important tool for this purpose, and various examples of models for the simulation of drug delivery from SCLs to the tear film and aqueous humor are available in literature [12–15]. However, a comprehensive model of the eye pharmacokinetics after wearing a therapeutic SCL, including drug transport from the anterior to the posterior segment, the barrier properties of the various ocular tissues and the eye physiology, is still missing.

**Fig. 1:**
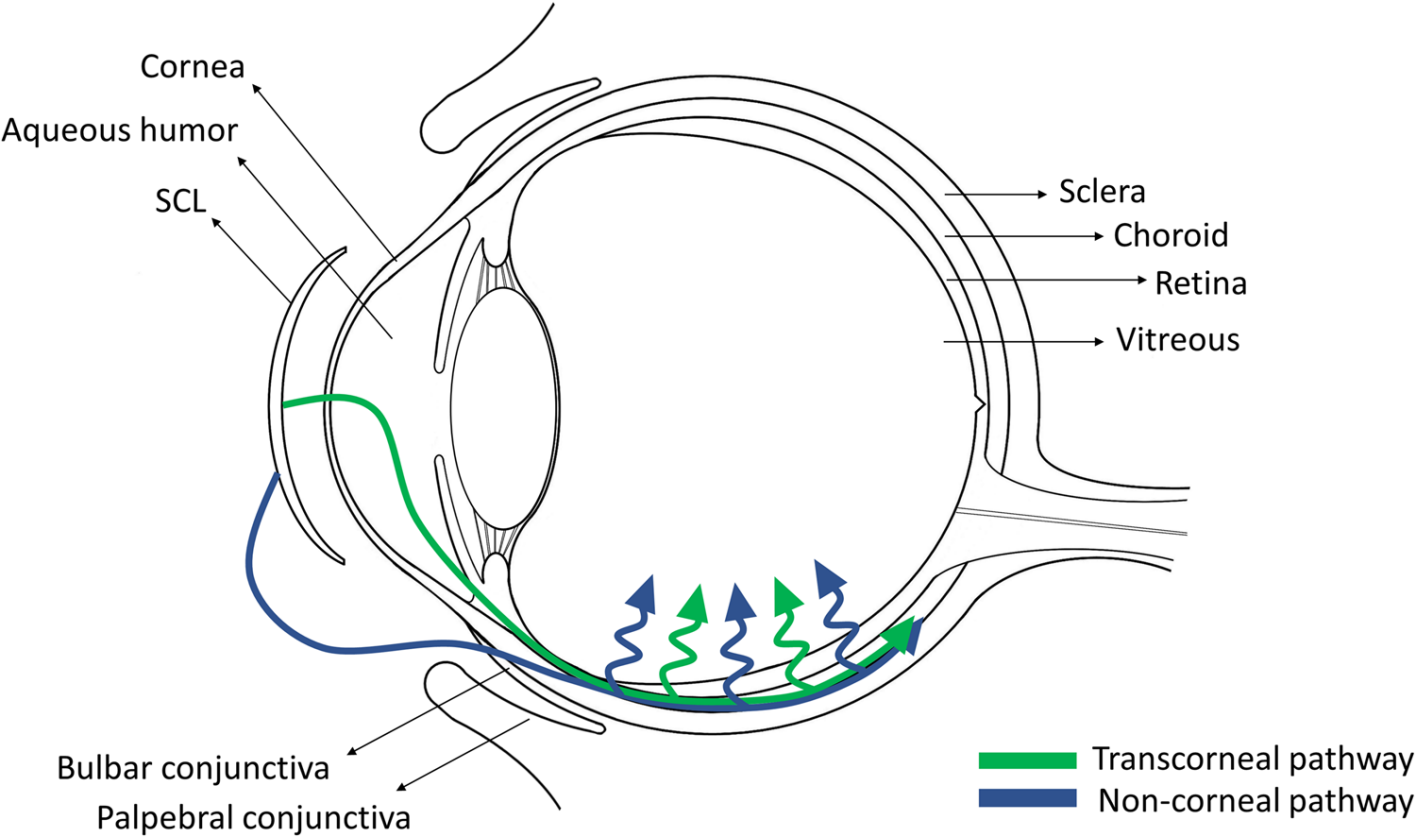
Schematic representation of the eye anatomy, SCL placement and main drug transport pathways to the posterior segment: transcorneal and non-corneal routes.

Herein, a mathematical model simulating drug diffusion from SCLs and drug accumulation over time in the ocular tissues of both the anterior and posterior segment of the eye (i.e. aqueous humor, sclera and choroid, vitreous and retina) is proposed. Two main pathways were identified for drug transport from the SCL to the choroid, and subsequently to the retina: transcorneal and non-corneal transport (Fig. 1). In transcorneal transport, drug diffuses to the aqueous humor during prolonged SCL wearing, and is then transported to the choroid and sclera by the uveoscleral outflow [16]. In non-corneal transport, drug diffuses from the SCL to the tear film, and then permeates across the bulbar conjunctiva and sclera to reach the choroid [17]. In both cases, after reaching the anterior choroid, the drug can rapidly reach the posterior segment of the eye via pressure-driven convection [18] and diffuse to the retina along the path.

The model parameters consisted in the barrier properties of the ocular tissues to drug diffusion and their drug partition coefficients, which can be determined via *ex vivo* studies, and the drug partition coefficient and diffusivity through the SCLs, obtained *in vitro*. The validation of the predicted results was performed by comparison with literature data on the *in vivo* drug delivery of from SCLs to the anterior and posterior segment of the eye.

## Methods

### Model Description

The abbreviations used in the model are defined in Table I for clarity. The drug concentrations in the contact lens, tears and ocular tissues can be computed by solving the mass balance equations along with appropriate boundary conditions. The model considers drug concentrations in seven different compartments: contact lens (*C*_*SCL*_), tears (*C*_*t*_), sclera-choroid (*C*_*ScCh*_), retina (*C*_*Ret*_), vitreous humor (*C*_*Vit*_), aqueous humor (*C*_*Aq*_) and corneal epithelium (*C*_*Ep*_). The sclera and choroid are lumped into a single compartment with weighted average partition coefficient, as there is no significant transport barrier between these tissues [19, 20]. The concentration in each tissue is considered independent of the position, as the drug distribution is assumed to be homogeneous. It is further assumed that the mass transfer resistance offered by the corneal endothelium is negligible [21], thus allowing for the stroma to be lumped together with the aqueous humor in the model, while the corneal epithelium is treated as a separate compartment (*C*_*Ep*_). Based on these assumptions, the following equations are obtained for the seven compartments:

**Table I:** Definitions of the Model Components. ^(a)^Parameters’ Values Reported in Literature; ^(b)^Calculated as in Eq. 14; ^(c)^Assumed Equal to 1; ^(d)^Estimated for the Specific Drug from Literature Values, Based on the Drug’s Lipophilicity, and Fine-Tuned During the Model Validation; ^(e)^Obtained from the Drug Amount Loaded in the SCL and/or the Release Profile

Physiological parameters^(a)^					
A_Bulb_	(cm^2^)	Exposed area of bulbar conjunctiva	Q_UvSc_	(mL/s)	Uveoscleral outflow
A_Cornea_	(cm^2^)	Area of the cornea	Q_Vit-Aq_	(mL/s)	Vitreous-aqueous drug elimination pathway
A_Globe_	(cm^2^)	Area of the eye globe	V_Aq_	(mL)	Volume of the aqueous humor
A_Palp_	(cm^2^)	Exposed area of palpebral conjunctiva	V_Ret_	(mL)	Volume of the retina
Clearance_ScCh_	(mL/s)	Drug clearance in the sclera-choroid through choroidal blood flow	V_ScCh_	(mL)	Volume of the sclera and choroid
Q_Aq_	(mL/s)	Aqueous humor renovation rate	V_t_	(mL)	Volume of the tears
Q_Drain_	(mL/s)	Tears drainage	V_Vit_	(mL)	Volume of the vitreous humor
Drug-dependent parameters					
F	(%)	Drug bioavailability in the aqueous humor^(b)^	K_t_	–	Partition coefficient of the drug in tears with respect to buffer^(c)^
K_Aq_	–	Partition coefficient of the drug in the aqueous humor with respect to buffer^(c)^	K_Vit_	–	Partition coefficient of the drug in the vitreous with respect to buffer^(c)^
K_Ep/Aq_	–	Partition coefficient of the drug in the corneal epithelium with respect to aqueous humor^(d)^	P_Conj_	(cm/s)	Drug permeability across the conjunctiva^(a)^
K_Ep/t_	–	Partition coefficient of the drug in the corneal epithelium with respect to tears^(d)^	P_Ret-Vit_	(cm/s)	Permeability of the inner limiting membrane^(d)^
K_SCL_	–	Partition coefficient of the drug in the contact lens with respect to buffer^(e)^	P_ScCh-Ret_	(cm/s)	Permeability of the retinal pigment epithelium^(a)^
K_Ret_	–	Partition coefficient of the drug in the retina with respect to buffer^(d)^	P_t-Aq_	(cm/s)	Permeability of the corneal epithelium^(d)^
K_ScCh_	–	Partition coefficient of the drug in the sclera-choroid with respect to buffer^(d)^			
SCL-dependent parameters					
A_SCL_	(cm^2^)	Area of the contact lens	y	(cm)	Spatial coordinate of the contact lens in transverse direction
D_SCL_	(cm^2^/s)	Drug diffusivity in the contact lens^(e)^	M_Released_	(ng)	Drug amount released by the contact lens^(e)^
H	(cm)	Thickness of the contact lens	R	(ng/s)	Drug release rate from the contact lens^(e)^
Exposure measurements					
C_Aq_	(ng/mL)	Drug concentration in the aqueous humor	C_ScCh_	(ng/g)	Drug concentration in the sclera and choroid
C_Ep_	(ng/g)	Drug concentration in the corneal epithelium	C_t_	(ng/mL)	Drug concentration in tears
C_SCL_	(ng/g)	Drug concentration in the contact lens	C_Vit_	(ng/mL)	Drug concentration in the vitreous humor
C_Ret_	(ng/g)	Drug concentration in the retina

1 $$\frac{\partial {C}_{SCL}}{\partial t}={D}_{SCL}\frac{\partial^2{C}_{SCL}}{\partial {y}^2}$$

2 $${V}_t\frac{d{C}_t}{dt}=-{D}_{SCL}\frac{\partial {C}_{SCL}}{\partial y}\left(y=0\right){A}_{SCL}-{Q}_{Drain}{C}_t-{P}_{Conj}{A}_{Palp}{C}_t-{P}_{Conj}{A}_{Bulb}\left(\frac{C_t}{K_t}-\frac{C_{ScCh}}{K_{ScCh}}\right)$$

3 $${V}_{ScCh}\frac{d{C}_{ScCh}}{dt}={Q}_{UvSc}{C}_{Aq}-{Q}_{UvSc}\frac{C_{ScCh}}{K_{ScCh}}+{P}_{Conj}{A}_{Bulb}\left(\frac{C_t}{K_t}-\frac{C_{ScCh}}{K_{ScCh}}\right)-{Clearance}_{ScCh}{C}_{ScCh}-{A}_{Globe}{P}_{ScCh- Ret}\left(\frac{C_{ScCh}}{K_{ScCh}}-\frac{C_{Ret}}{K_{Ret}}\right)$$

4 $${V}_{Ret}\frac{d{C}_{Ret}}{dt}={A}_{Globe}{P}_{ScCh- Ret}\left(\frac{C_{ScCh}}{K_{ScCh}}-\frac{C_{Ret}}{K_{Ret}}\right)-{A}_{Globe}{P}_{Ret- Vit}\left(\frac{C_{Ret}}{K_{Ret}}-\frac{C_{Vit}}{K_{Vit}}\right)$$

5 $${V}_{Vit}\frac{d{C}_{Vit}}{dt}={A}_{Globe}{P}_{Ret- Vit}\left(\frac{C_{Ret}}{K_{Ret}}-\frac{C_{Vit}}{K_{Vit}}\right)-{Q}_{Vit- Aq}{C}_{Vit}$$

6 $${V}_{Aq}\frac{d{C}_{Aq}}{dt}={Q}_{Vit- Aq}{C}_{Vit}+{A}_{Cornea}{P}_{t- Aq}\left(\frac{C_{SCL}\left(y=H\right)}{K_{SCL}}-{C}_{Aq}\right)-{Q}_{Aq}{C}_{Aq}$$

7 $${C}_{Ep}=\frac{\left({K}_{Ep/ Aq}{C}_{Aq}+{K}_{Ep/t}{C}_t\right)}{2}$$

**Fig. 2:**
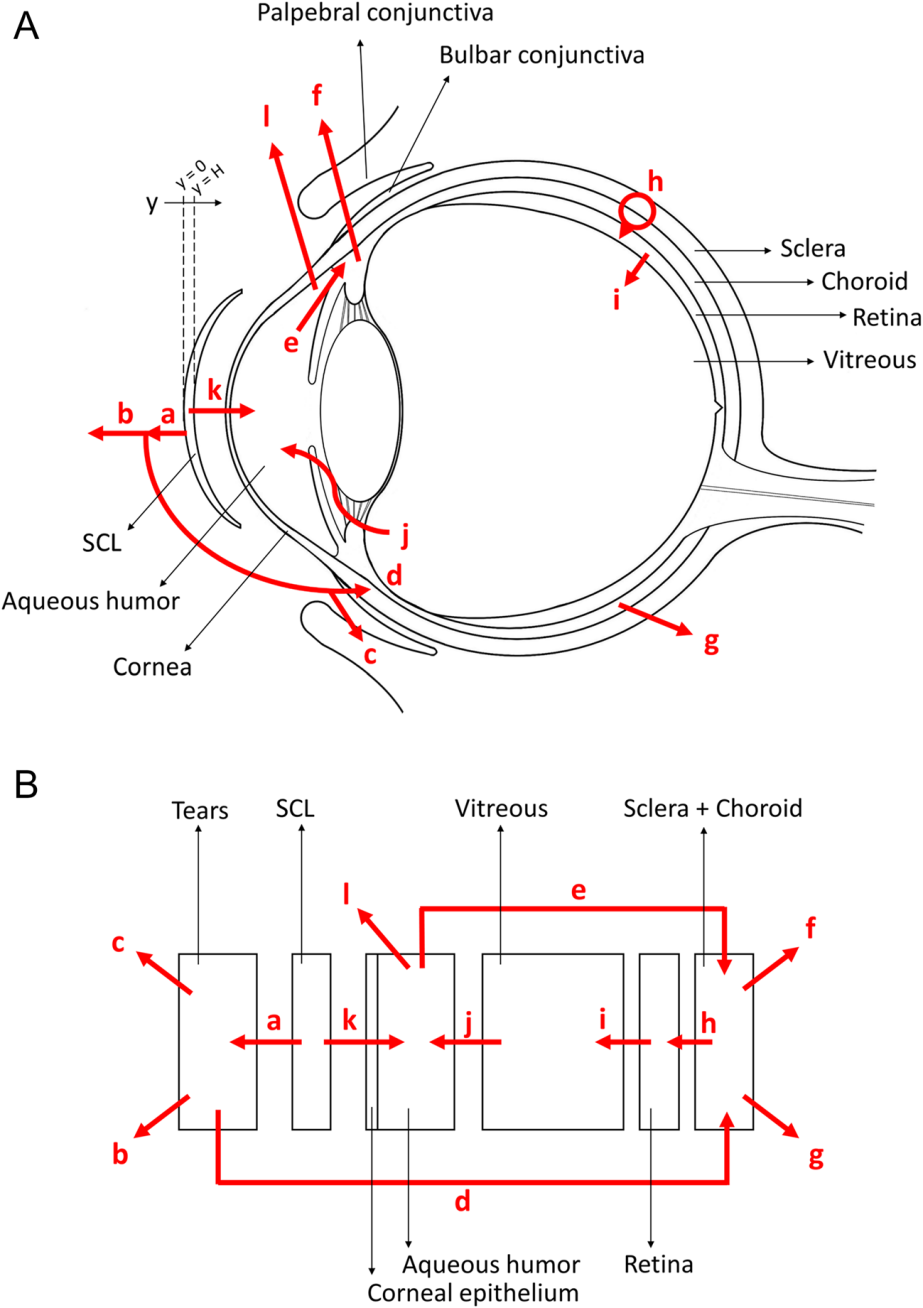
Schematic representation of the eye anatomy, SCL placement and drug transport pathways (red arrows a-l) (**A**); mathematical model design with transport pathways (red arrows a-l) (**B**).

Equation 1 is a partial differential equation because the concentration in the contact lens depends both on time (t) and position (y), the latter referring to the transversal direction of the SCL, with y = 0 at the anterior lens-tears interface, and y = H at the lens-post lens tear film (POLTF) interface. The lens thickness H is assumed to be homogeneous. All other mass balance equations are ordinary differential equations because the spatial variations in tissue concentrations are neglected. The left-hand side of each of the mass balances is the net accumulation in the tissue, while the right-hand side includes the rates of all transport pathways that bring drug in or take drug out of the tissues (Fig. 2, arrows a-l). The rate of transport across any membrane is the product of the exposed area (A), permeability (P), and the net driving force (i.e., the concentration difference). The flows between compartments are indicated as Q. In Eq. 2, the drug accumulation in tears results from the sum of four transport pathways: drug in from lens (a), drug out via drainage (b), drug out via palpebral conjunctiva (c) and drug out via bulbar conjunctiva (d). The terms on the right-hand side of sclera-choroid mass balance (Eq. 3) represent drug in from aqueous humor via uveoscleral outflow (e), drug clearance due to seepage of the uveoscleral outflow into the orbit vasculature (f) [22], drug in from tears across bulbar conjunctiva (d), clearance through choroidal blood flow (g) and drug out into retina through retina pigment epithelium (h). The two transport pathways of Eq. 4 represent drug into retina from sclera-choroid (h) and drug out into vitreous humor (i). The two terms in Eq. 5 represent drug into vitreous from retina (i) and drug out into aqueous (j). The three terms in Eq. 6 represent drug into the aqueous from vitreous (j), drug in from the contact lens across the corneal epithelium (k) and drug out by aqueous humor renovation (including drug out into the vasculature through trabecular meshwork/Schlemm canal pathway (l) and uveoscleral outflow (e) [22, 23]). Finally, the corneal epithelium concentration (Eq. 7) is assumed to vary linearly across its thickness and to be in equilibrium with tears on one side and with the aqueous humor on the other.

Equation 1 requires the following boundary conditions:

8 $${C}_{SCL}\left(y=0\right)={K}_{SCL}{C}_t$$

9 $$-{D}_{SCL}\frac{\partial {C}_{SCL}}{\partial y}\left(y=H\right)={A}_{Cornea}{P}_{t- Aq}\left(\frac{C_{SCL}\left(y=H\right)}{K_{SCL}}-{C}_{Aq}\right)$$

which imply equilibrium between tears and the anterior side of the lens (Eq. 8) and equality between the flux out from the lens into the POLTF and the flux across the epithelium into aqueous humor (Eq. 9). At *t = 0*, all tissue concentrations are zero and the concentrations in the anterior and posterior surfaces of the SCL are known based on the loading conditions.

### Model Validation

Previous studies [9, 10, 24–27] reporting *in vivo* data on the drug concentration in the ocular tissues after the application of a therapeutic SCL were collected to validate the model predictions. Rabbit models are the current standard for *in vivo* studies involving contact lenses, with well-known geometrical and physiological parameters of the rabbit eye available in literature. While most of the studies on ocular drug distribution after wearing a SCL only analyze drug concentration in the internal ocular tissues at a single time point [10, 27], a study by Ross *et al.* [9] reports the concentration of dexamethasone, a commonly administered anti-inflammatory drug, in both the anterior and posterior segment of rabbit eyes over a 7-days period. Such data currently constitutes the only available information on ocular drug distribution from contact lenses over time.

Despite the mathematical model being originally developed considering drug delivery from the SCLs as a diffusion-controlled phenomenon (Eqs. 1 and 2), the type of SCL used in Ross’ study is a sandwich device encapsulating a degradable drug-eluting polymer film between two hydrogel layers. Such lens is a complex system, and the modelling of its release profile requires both the degradation of the polymer film and the subsequent diffusion across the hydrogel shell to be considered. Development of the matrix degradation model requires multiple parameters that are not available and so a modified approach was adopted by directly using *in vitro* release data to estimate release from the lens in the eye. The drug released from the lens could diffuse both towards the tears and towards the cornea. A model based on diffusion through the lens with appropriate boundary condition at the interface with tears can predict the fraction of drug that diffuses in both directions. Since the detailed transport model through the lens is not utilized, a fitting parameter is needed to reflect the portion of drug released from the lens that diffuses towards the cornea. Equations 2 and 6 were therefore modified as in Eqs. 10 and 11, where R is the *in vitro* drug release rate from the SCL and F is the drug bioavailability in the aqueous humor (obtained from *in vivo* data)*.*

10 $${V}_t\frac{d{C}_t}{dt}=\left(1-F\right)\ R-{Q}_{Drain}{C}_t-{P}_{Conj}{A}_{Palp}{C}_t-{P}_{Conj}{A}_{Bulb}\left(\frac{C_t}{K_t}-\frac{C_{ScCh}}{K_{ScCh}}\right)$$

11 $${V}_{Aq}\frac{d{C}_{Aq}}{dt}={Q}_{Vit- Aq}{C}_{Vit}+F\ R-{Q}_{Aq}{C}_{Aq}$$

The drug release from the SCL over time, *f,* was obtained by fitting the experimental *in vitro* data to an exponential curve (Eq. 12). Then, R was calculated as in Eq. 13, where M_Released_ is the total amount of drug released by the SCL and T is the time constant obtained by curve fitting.

12 $$f={M}_{Released}\left(1-{e}^{-t/T}\right)$$

13 $$R=\frac{df}{dt}=\frac{M_{Released}}{T}\ {e}^{-t/T}$$

F was then calculated as follows:

14 $$F\%=\frac{\int_0^{t_{max}}{Q}_{Aq}\times {C}_{Aq}\ dt}{M_{Released}}\times 100=\frac{Q_{Aq}\times {AUC}_{\left({C}_{Aq}\right)}}{M_{Released}}\times 100$$

Where Q_Aq_ and C_Aq_ are the aqueous humor renovation rate and drug concentration, respectively. The area under the curve (AUC) of C_Aq_ over time is obtained by fitting the *in vivo* data to an exponential curve and integrating up to C_Aq_ = 0 (Supplementary Fig. S1).

### Sensitivity Analysis

The variations in the concentration-time curves of the various compartments (i.e., vitreous humor, aqueous humor, sclera-choroid, retina and tears) with different input values were registered to investigate the sensitivity of the model. The anatomical parameters of the eye were considered constant and are reported in Table II.

**Table II:** Anatomical Parameters of the Rabbit Eye Used in the Simulations

Parameter	Value	Ref.
V_t_	7E-03 mL	[28]
V_Vit_	1.7 mL	[29]
V_ScCh_	0.361 mL	Thickness of sclera [30] and choroid [31] x A_globe_
V_Ret_	0.086 mL	Thickness of retina [29] x A_globe_
V_Aq_	0.325 mL	[29]
A_Globe_	8.6 cm^2^	[32]
A_Palp_	14 cm^2^	[33]
A_Bulb_	3 cm^2^	[33]

A local sensitivity analysis was performed by increasing or decreasing by 10% one parameter at a time (i.e., barrier properties of the tissues, physiological transport pathways and partition coefficients) and evaluating the impact of the variation on the AUC and maximum drug concentration (C_max_) of each concentration-time curve.

The visualization of uncertainty was then obtained by simultaneously varying all input values, except for the anatomical parameters. Each parameter was randomized in the ±10% range from its nominal value (N = 400 runs).

### Software

The model implementation and the sensitivity analysis were performed in MATLAB - version R2019b (The MathWorks Inc., Natick, MA, USA). Results were plotted on Prism 8.0.1 software (GraphPad, San Diego, CA, USA).

## Results and Discussion

### Model Validation

*In vivo* data by Ross *et al.* [9] were used for model validation by comparison with the simulated values over time. In Ross’ study, dexamethasone was detected over a 7-days span in the aqueous humor, choroid and sclera, retina and vitreous humor of rabbits after SCL wearing. The anatomical parameters of the eye used in the simulation are reported in Table II. The drug- and SCL-specific parameters (i.e., drug permeability P across the ocular barriers, the partition coefficient K of the drug with the tissues and the aqueous humor bioavailability F %) are reported in Table III. The physiological parameters of the rabbit eye are reported in Table IV.

**Table III:** Drug- and SCL-Specific Parameters Used in the Simulation to Validate the Proposed Model with the Reported *In Vivo* Data [9]

Parameter	Value	Ref
P_Conj_	2.50E-06 cm/s	[34]
P_ScCh-Ret_	10E-06 cm/s	[31]
P_Ret-Vit_	13E-06 cm/s	Considered 30% higher than P_ScChRet_ [35]
K_ScCh_	15	Adapted for dexamethasone from previously reported data [36]
K_Ret_	10	Adapted for dexamethasone from previously reported data [36]
F	2.08%	Calculated as in Eq. 14

**Table IV:** Physiological Parameters of the Rabbit Eye Used in the Simulation

Parameter	Value	Ref
Q_UvSc_	0.176 μl/min	[37]
Q_Vit-Aq_	0.19 μl/min	[35]
Q_Aq_	4.2 μl/min	[38]
Q_Drain_	0.5 μl/min	[39]
K_Vit_	1	Assumed equal to buffer
K_Aq_	1	Assumed equal to buffer
K_t_	1	Assumed equal to buffer
Clearance_ScCh_	1 mL/min	[40]

**Fig. 3:**
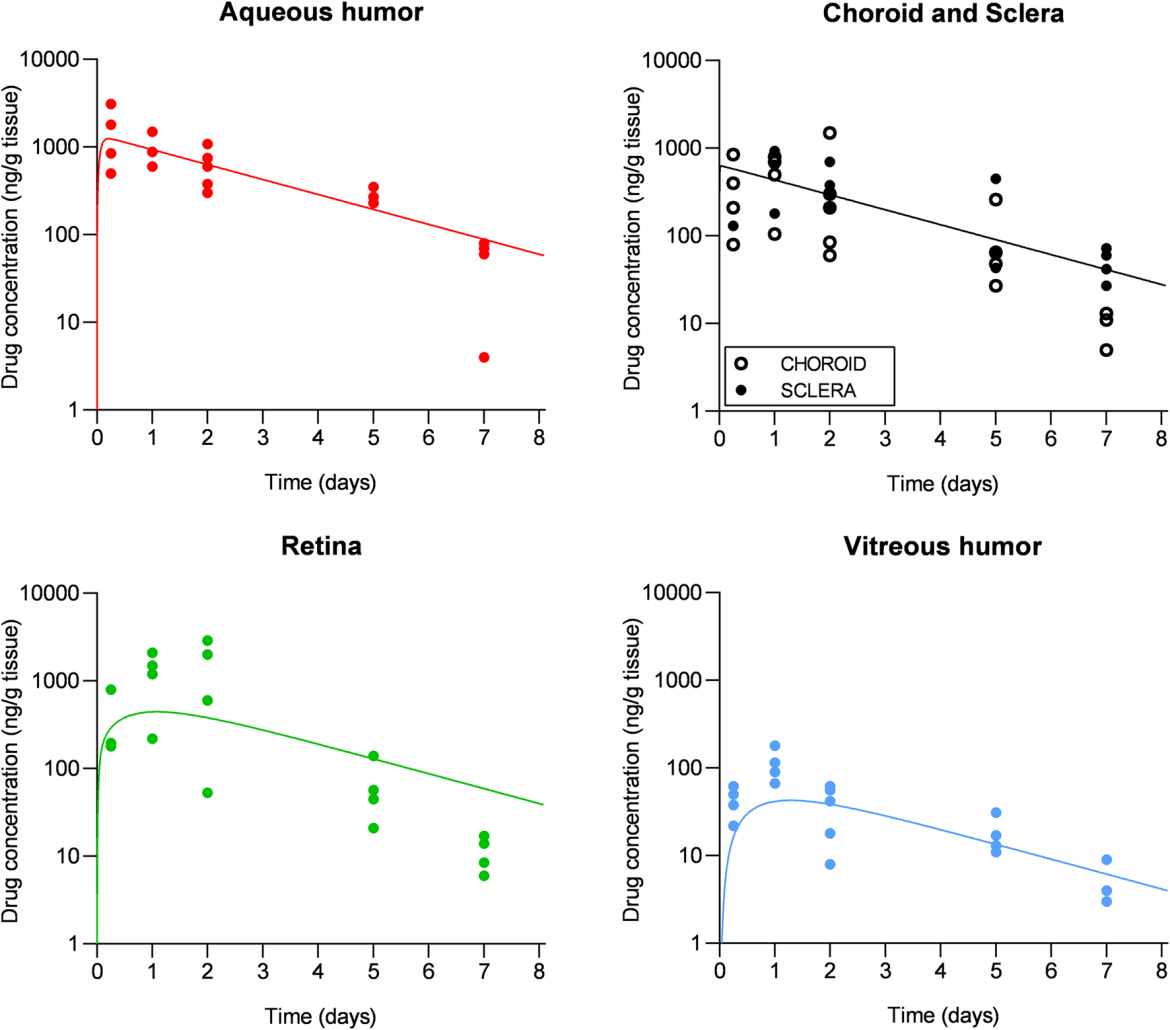
Comparison between experimental data obtained in rabbits (points) [9] and model prediction (curves) of dexamethasone concentration in the ocular tissues after application of a drug-loaded contact lens.

A comparison between experimental and predicted values is shown in Fig. 3. A large variability was observed in the reported *in vivo* concentrations, possibly due to the natural differences between the tested animals and to the error associated with drug extraction from biological tissues. Nonetheless, the model was able to predict experimental data in both the anterior and posterior segment tissues and provided a good estimate of the drug concentration over time both in the long term and short term.

The experimental values obtained for the retina are predicted by the mathematical model in the first days of treatment, but overestimated in the long term (i.e., after 5 and 7 days). The high metabolic activity of the retina pigmented epithelium may play a role in the faster clearance kinetics of dexamethasone in the tissue [41]. In fact, higher levels of cytochrome P450 enzymes and drug transporters (which regulate drug metabolism and transport in the liver, small intestine and kidneys) were detected in the retina/choroid when compared with other ocular tissues [42]. However, Ross’ study constitutes the only available data on drug concentrations in the retina over time. The future availability of *in vivo* data on the delivery of different drugs from SCLs to the posterior segment will allow for a more extended validation of the model for the prediction of drug concentration in the retina.

**Fig. 4:**
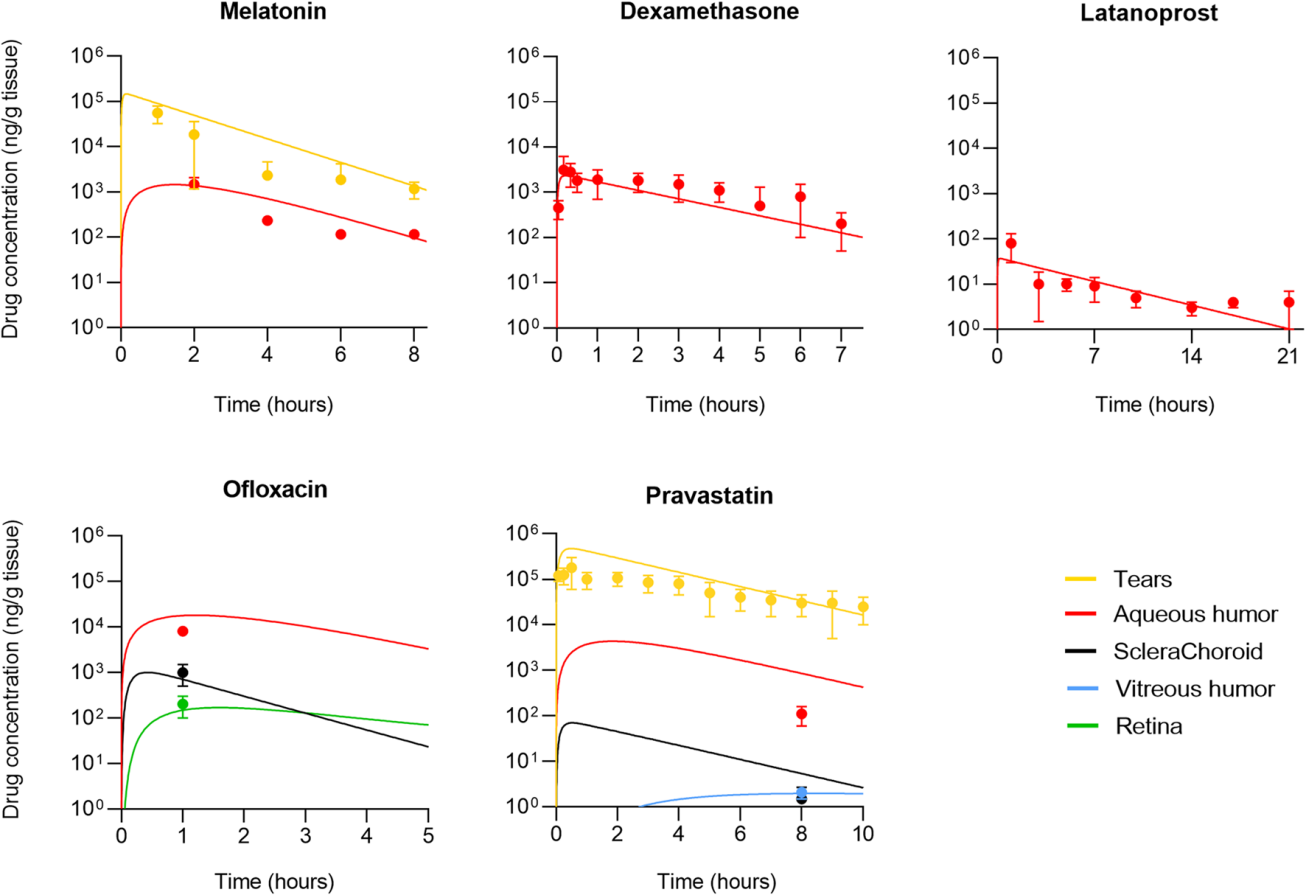
Comparison between experimental data obtained in rabbits (points: melatonin [24], dexamethasone [25], latanoprost [26], ofloxacin [27] and pravastatin [10]) and model prediction (curves) of drug concentration in the tears and/or ocular tissues after application of a drug-loaded contact lens.

Comparisons of the model predictions to other reported *in vivo* data [10, 24–27] are shown in Fig. 4. The selected drug- and SCL-specific input values for the simulations are reported in Supplementary Table S1. The model results were considered representative of the experimental data and provided a prediction of the drug concentration over time both in the long term (dexamethasone and latanoprost release) and short term (melatonin, ofloxacin and pravastatin release).

All the selected studies reported the drug concentration in the aqueous humor, which could be predicted by the mathematical model with different degrees of error. For what concerns the drug distribution in the internal ocular tissues, the concentration of ofloxacin was measured in the retina and sclera [27], while amounts of pravastatin were quantified in the sclera and vitreous humor [10]. The model predicted well the experimental data in the internal tissues, but, for these data sets, an evaluation of the accuracy of prediction over time was not possible due to the availability of a single time point.

### Sensitivity Analysis

**Fig. 5:**
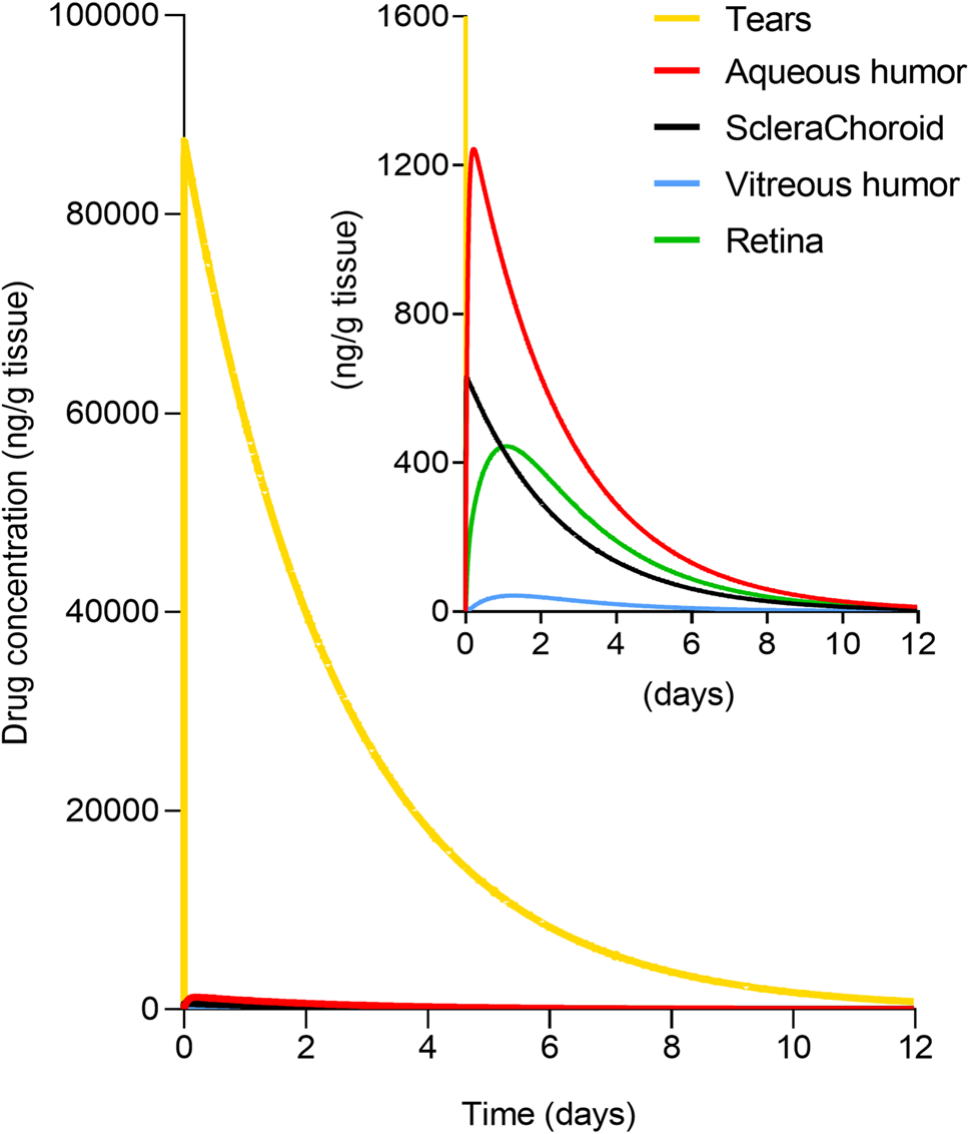
Model output: drug concentration in the ocular tissues over time, normalized per tissue or fluid weight. A zoomed representation is shown in the figure inset.

**Fig. 6:**
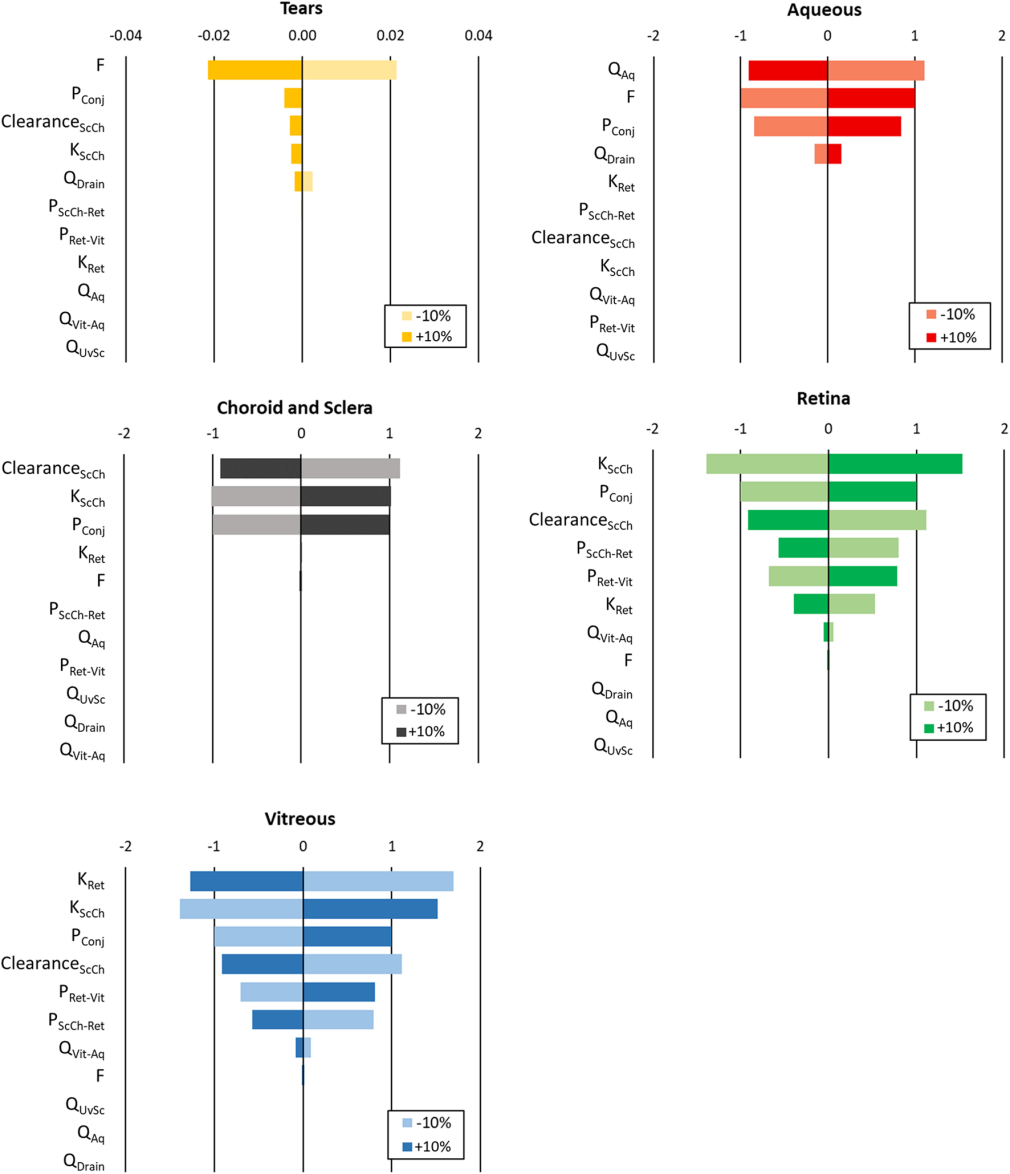
Percentage variation of the AUC of the predicted drug concentration in the ocular tissues over time with respect to the percentage variation of each input parameter value (Δ_AUC (%)_ /ǀΔ_parameter (%)_ǀ).

**Fig. 7:**
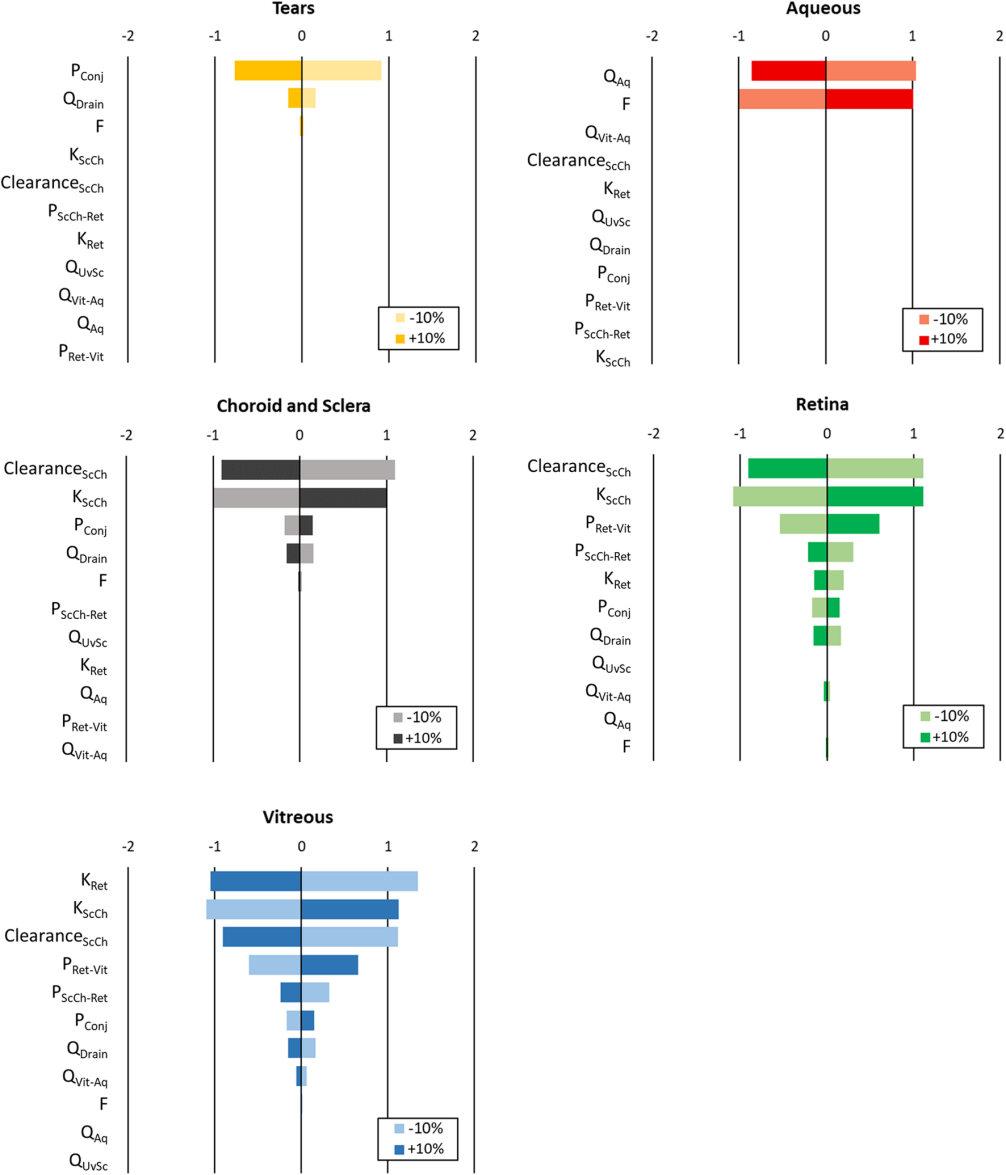
Percentage variation of the predicted maximum drug concentration C_max_ in the ocular tissues with respect to the percentage variation of each input parameters value (Δ_Cmax (%)_ /ǀΔ_parameter (%)_ǀ).

The development of a mathematical model involving the full eye allows to evaluate the role of each parameter on drug accumulation in each ocular tissue, and the relative importance of these parameters in drug transport to the posterior segment. The model prediction of the drug concentration in the tears and ocular tissues over time, obtained with the input parameters values reported in Section 3.1, is shown in linear scale in Fig. 5. Results of the local sensitivity analysis on the AUC and C_max_ are reported in Figs. 6 and 7, respectively.

As expected, the AUC and C_max_ of the deeper tissues (i.e., the retina and vitreous humor) are dependent on a higher number of input parameters if compared to the external layers of the eye.

The drug permeability across the ocular tissues, and in particular the conjunctival permeability P_conj_, appears to be an influential factor in the determination of the AUC values of all tissues (Fig. 6). The AUC of the retina and vitreous humor are also determined by the permeability of the retinal pigment epithelium (P_ScCh-Ret_) and of the inner limiting membrane (P_Ret-Vit_). The AUC of the vitreous humor, sclera-choroid and retina is strongly impacted by the affinity of the drug with the tissues, represented by the partition coefficients K_ScCh_ and K_Ret_, and by the clearance of the sclera-choroid due to choroidal blood flow.

Similarly to what was observed from the AUC results, the partition coefficients (K_ScCh_ and K_Ret_) and the clearance of the sclera-choroid determine the C_max_ in the vitreous, sclera-choroid and retina (Fig. 7). The drug permeability values across the tissues (P_Conj_, P_Ret-Vit_ and/or P_ScCh-Ret_) are significant in the determination of the C_max_ in all tissues with the exception of the aqueous humor. The C_max_ of the aqueous humor is instead determined by its renovation rate Q_Aq_ and by the bioavailability F, which implicitly includes information on the drug hydrophilicity and permeability through the cornea. The influence of the ocular flows Q is higher on the variation of C_max_ in all tissues, if compared to variations in the AUC. Variations in Q_Drain_, in fact, reflect not only on the C_max_ of the tear film, but also of the sclera-choroid, retina and vitreous.

The visualization of uncertainty is shown in Supplementary Fig. S2 for all tissues. The concentration profiles obtained with the input values reported in Section 3.1 are represented in a cloud of curves obtained with randomized input values in the ±10% variation interval from the nominal values. The uncertainty is limited to the proximity of the curves’ peak, and is not increasing over time, thus describing an overall robust model.

### Potential and Limitations of the Model

A mathematical model was proposed to better understand the ocular pharmacokinetics after the application of a therapeutic SCL. With a simplified physiology-based approach, the model includes the most important drug transport pathways and was validated by comparison with *in vivo* data available in the literature. The model allowed for the study of the relative importance of the involved drug transport parameters (i.e., drug permeability in the tissues, ocular flow rates and partition coefficients of the drug in the tissues), and constitutes an important tool for the identification of the most critical barriers to the treatment of the posterior segment by topical drug administration. As such, it could be used for the strategic design of drug delivery technologies focused on overcoming these barriers and increase the efficiency of delivery.

The model was specifically developed for SCLs. Despite this, it offers a comprehensive view of the drug transport pathways in the eye and could be easily tuned to account for the use of other drug delivery devices. As an example, by changing the drug source location in the equations, it could be possible to model the ocular drug distribution over time from an intracameral or intravitreal implant, as well as from a drug-loaded intraocular lens or episcleral implant.

Some limitations of the model, however, need to be considered. Firstly, only a few *in vivo* studies [9, 10, 27] report the drug concentration in the posterior segment of the eye after wearing a therapeutic SCL, thus excluding the possibility of an extensive model validation. Among these studies, only Ross *et al.* [9] analyzed more than one time point, therefore allowing the comparison with the predicted drug concentration curves over time. Furthermore, the complex SCL system of Ross’ study required the introduction of a fitting parameter obtained from *in vivo* data (F), which could be overcome in the future by the possibility of validation with studies involving pure diffusion-controlled drug delivery from SCLs. As a result, the model would be independent on *in vivo* data and could be used as a predictive tool, able to provide an estimate of the duration of the therapeutic efficacy after the application of a SCL and simulate the drug concentration over time in the ocular tissues without need of animal testing. Mathematical simulations, in fact, are useful not only to clarify the ocular drug pathways and their relative importance in drug delivery, but also to reduce the gap between *in vitro* studies and clinical investigation, allowing for a fast, simple and inexpensive evaluation of the efficacy of SCLs in the early stage of product development and shortening the path towards the commercialization of such drug delivery devices. The high cost and ethical issues associated with animal testing [43] are obstacles to SCLs optimization based on their *in vivo* performance. *In vitro* characterization, on the other hand, may be used in the early stages of design but is generally insufficient to provide adequate predictions of the device efficacy [44].

The precision of the model validation was affected by the high standard deviation observed in the *in vivo* experimental data, which is mainly due to inter-animal variability and to difficulties in the handling and analysis of ocular tissues. The intrinsic variability in animal tissues also influences the determination of the values of the input parameters: while the physiological parameters of the eye could be considered as fixed values, the barrier properties of the tissues (described in the model by the permeability values P and partition coefficients K) still require experimental data, usually obtained *ex vivo*. *Ex vivo* tests allow for the estimation of the drug-tissue interactions to some extent, but are still subjected to the variability associated with biological tissues, as demonstrated by the wide range of values reported by different authors for the same drug-tissue combination (e.g., P_app_ = 2-25 × 10^−6^ cm/s for dexamethasone across the excised cornea [45–47]). Moreover, as active mechanisms of drug transport may not be fully preserved in excised tissues [48], the correlation between *ex vivo* and *in vivo* data could be inaccurate. Despite these known limitations, *ex vivo* studies allow to reduce the use of *in vivo* tests, are currently the standard tool to obtain information on drug permeation across the tissues (especially in comparative studies) and, as opposed to *in vitro* cellular models, allow for the preservation of the natural tissue structure [49].

It is worth considering that the reported *in vivo* tests were performed with small drug molecules (e.g., 392.47 Da for dexamethasone). For this reason, it was not possible to verify the validity of the mathematical model for the delivery of macromolecules. Despite small drugs being the most commonly used in both research and clinical applications, the use of biopharmaceuticals have had a significant growth in ophthalmology with an associated increased research interest for alternative and more efficient delivery methods [50].

The use of permeability enhancers, prodrugs, drug carrier molecules or nanoparticles also gained interest in the last decade to improve drug delivery efficiency and therapeutic efficacy, especially in the treatment of the posterior segment of the eye. These strategies are not considered in the model, which assumes the properties of each tissue and drug to be constant over time and space. Modifications of the model are possible but would increase the complexity of the proposed transport mechanisms, would require validation for each specific drug transport method and extensive understanding of the drug/carrier behavior *in vivo*, which is generally not easily available.

## Conclusion

A mathematical model was developed to include the most important ocular drug transport pathways and tissues properties and help understanding the mechanisms of drug delivery from the anterior to the posterior segment of the eye after the application of a drug eluting SCL. The drug concentration in the tears, aqueous humor, sclera and choroid, retina and vitreous humor was predicted and results were validated by comparison with previously reported *in vivo* data. The sensitivity analysis identified the drug permeability across the conjunctiva (P_Conj_) and the partition coefficient of the drug in the sclera-choroid (K_ScCh_) as the most influential parameters in the determination of the drug accumulation over time in the majority of the ocular tissues. The model can also be tuned to estimate drug delivery to the posterior segment from other types of ocular drug delivery devices.

## Supplementary Information

ESM 1 (DOCX 437 kb)

## Data Availability

The datasets generated during and/or analyzed during the current study are available from the corresponding author on request.
