# Supplementary material for: A Physiology-Based Mathematical Model to Understand Drug Delivery from Contact Lenses to the Back of the Eye
Source: Pharm Res. 2023 Jul 27;40(8):1939–51. doi: 10.1007/s11095-023-03560-7 (PMC10447275; doi:10.1007/s11095-023-03560-7)
Supplement: Supplementary file 1 — (DOCX 437 kb) [file 11095_2023_3560_MOESM1_ESM.docx]

**
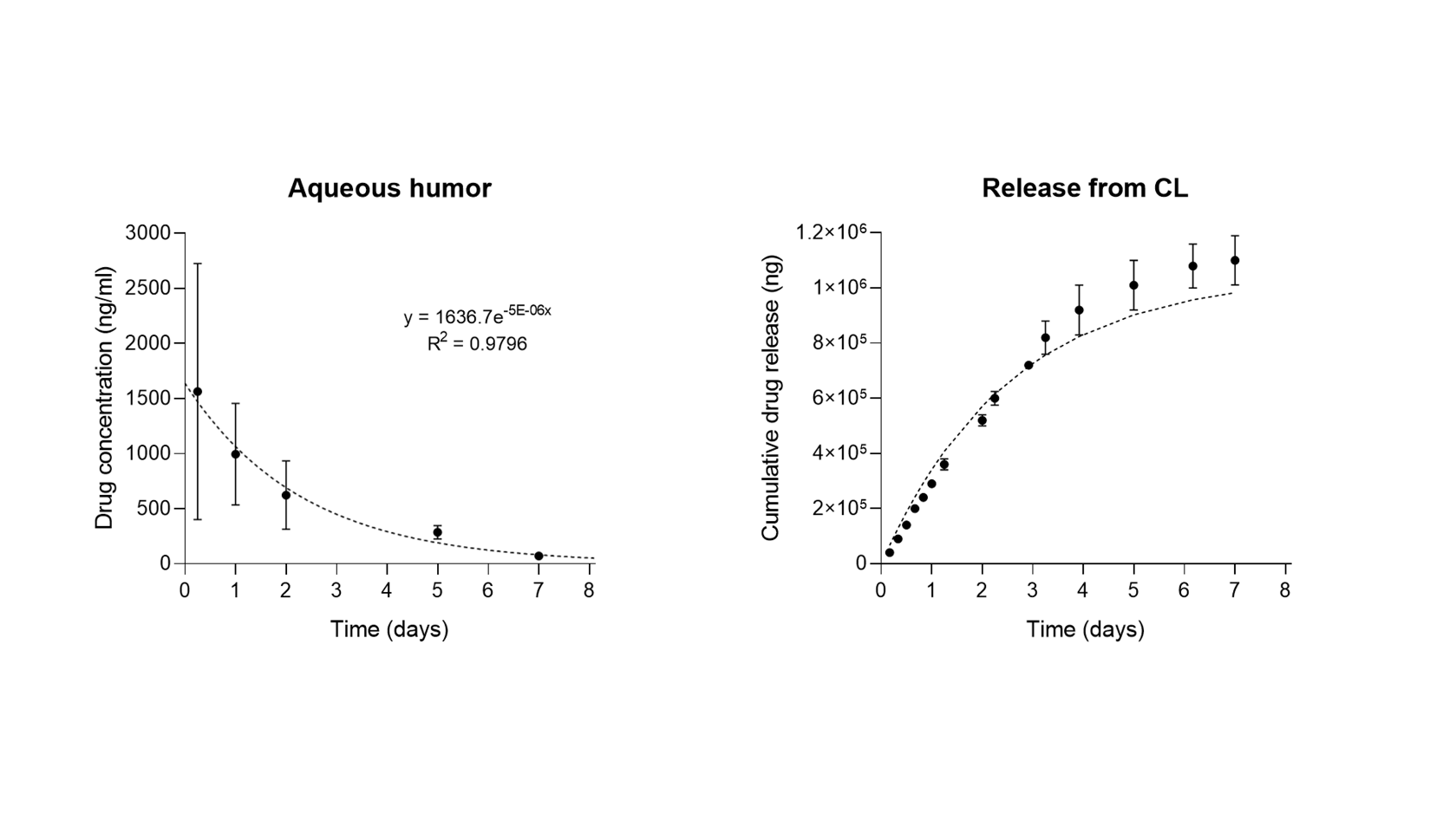
Supplementary Material**

*Supplementary Figure S1. To obtain the AUC of the drug concentration in the aqueous humor (C_aq_) over time, experimental data* [1] *were fitted to an exponential curve, then AUC was calculated integrating up to C_aq_ = 0.*

Supplementary Table S1. Drug- and SCL-specific parameters used in the simulations for each reported experimental data set.

| **Drug** | **Model input parameters** | | | | | | ***In vivo* data source** | **logD_7.4_** | |
| --- | --- | --- | --- | --- | --- | --- | --- | --- | --- |
|  | P_Conj_^a^  (cm/s) | P_ScCh-Ret_^b^  (cm/s) | P_Ret-Vit_^c^  (cm/s) | K_ScCh_^d^ | K_Ret_^d^ | F %^e^ |  | Value | Ref. |
| Dexamethasone | 2.5E-06 | 10E-06 | 13E-06 | 15 | 10 | 3.49 | [2] | 1.95 | [3] |
| Melatonin | 2.5E-06 | 10E-06 | 13E-06 | 15 | 10 | 2.93 | [4] | 1.19 | [5] |
| Latanoprost | 3.0E-06 | 10E-06 | 13E-06 | 17 | 11 | 2.08 | [6] | 4.28 | [7] |
| Ofloxacin | 2.5E-07 | 5E-06 | 7E-06 | 7 | 5 | 7.07* | [8] | -0.40 | [9] |
| Pravastatin | 2.5E-07 | 3E-06 | 4E-06 | 3 | 3 | 7.87* | [10] | -0.70 | [11] |
| ^a^Comparable to the values reported in literature [12] and adapted to the logD_7.4_ of each drug; ^b^Reported in previous literature for similar drugs [13]; ^c^Considered 30 % higher than P_ScChRet_ [14]; ^d^Comparable to previously reported data [15], scaled according to the logD_7.4_ of each drug and fine-tuned during the model validation; ^e^Calculated as in Equation 14, except when indicated (*): as the concentration of ofloxacin [8] and pravastatin [10] was measured *in vivo* in the aqueous humor at a single time point, F % was in these cases estimated based on the logD_7.4_ of each drug and fine-tuned during the model validation. | | | | | | | | | |

**
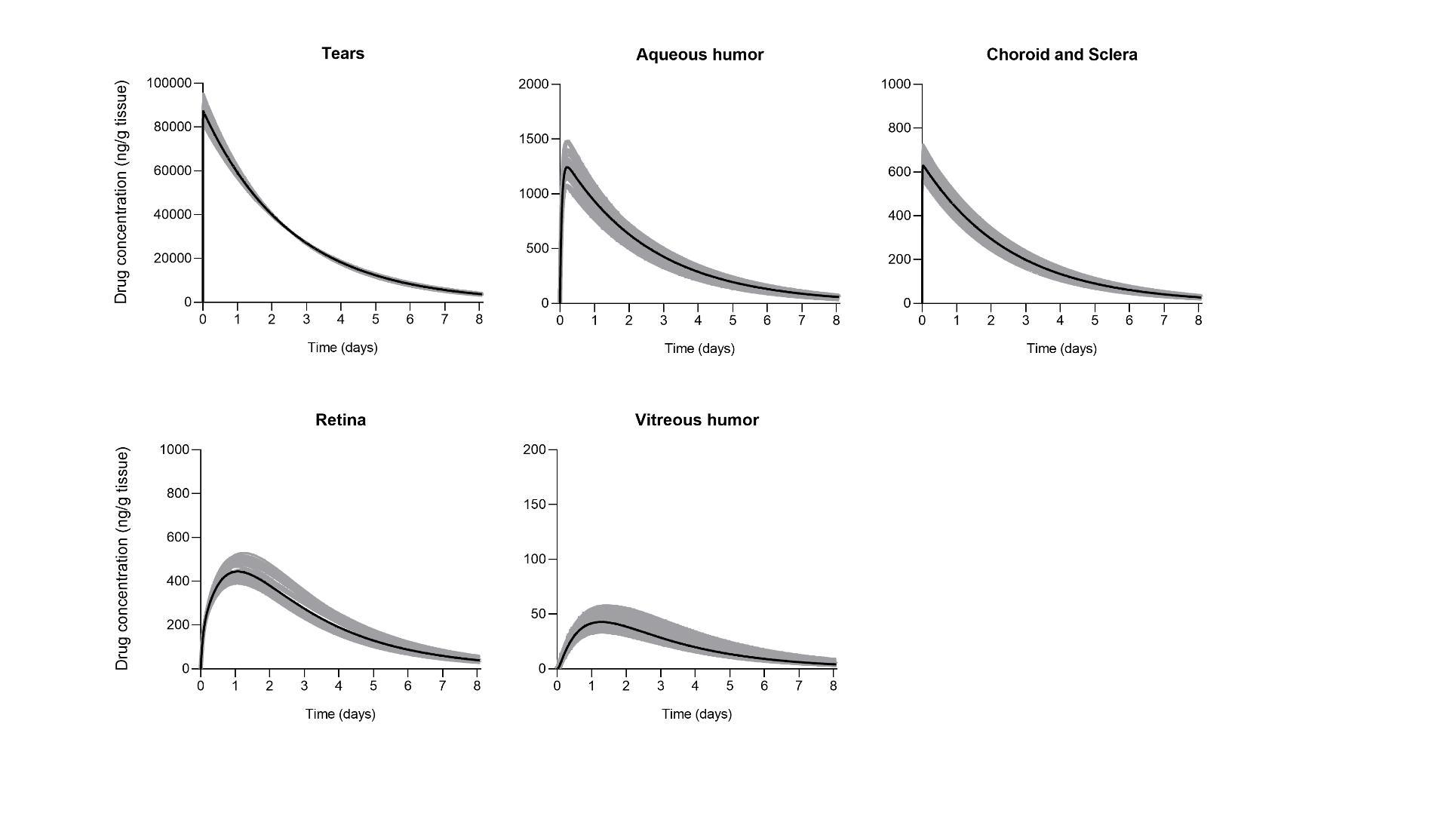
**

*Supplementary Figure S2. Visualization of uncertainty in the predicted drug concentration in the ocular tissues with randomized input values (grey curves) in the ± 10 % range from the nominal values (black curves).*

References

[1] Ross AE, Bengani LC, Tulsan R, Maidana DE, Salvador-Culla B, Kobashi H, et al. Topical sustained drug delivery to the retina with a drug-eluting contact lens. Biomaterials 2019;217:119285. https://doi.org/10.1016/j.biomaterials.2019.119285.

[2] Bengani LC, Kobashi H, Ross AE, Zhai H, Salvador-Culla B, Tulsan R, et al. Steroid-eluting contact lenses for corneal and intraocular inflammation. Acta Biomater 2020;116:149–61. https://doi.org/10.1016/j.actbio.2020.08.013.

[3] Naageshwaran V, Ranta VP, Toropainen E, Tuomainen M, Gum G, Xie E, et al. Topical pharmacokinetics of dexamethasone suspensions in the rabbit eye: Bioavailability comparison. Int J Pharm 2022;615:121515. https://doi.org/10.1016/j.ijpharm.2022.121515.

[4] Serramito M, Pereira-da-Mota AF, Carpena-Torres C, Huete-Toral F, Alvarez-Lorenzo C, Carracedo G. Melatonin-Eluting Contact Lenses Effect on Tear Volume: In Vitro and In Vivo Experiments. Pharmaceutics 2022;14:1019. https://doi.org/10.3390/pharmaceutics14051019.

[5] Ferreira MA, Azevedo H, Mascarello A, Segretti ND, Russo E, Russo V, et al. Discovery of ACH-000143: A Novel Potent and Peripherally Preferred Melatonin Receptor Agonist that Reduces Liver Triglycerides and Steatosis in Diet-Induced Obese Rats. J Med Chem 2021;64:1904–29. https://doi.org/10.1021/acs.jmedchem.0c00627.

[6] Ciolino JB, Stefanescu CF, Ross AE, Salvador-Culla B, Cortez P, Ford EM, et al. In vivo performance of a drug-eluting contact lens to treat glaucoma for a month. Biomaterials 2014;35:432–9. https://doi.org/10.1016/j.biomaterials.2013.09.032.

[7] Rodriguez-Aller M, Guinchard S, Guillarme D, Pupier M, Jeannerat D, Rivara-Minten E, et al. New prostaglandin analog formulation for glaucoma treatment containing cyclodextrins for improved stability, solubility and ocular tolerance. Eur J Pharm Biopharm 2015;95:203–14. https://doi.org/10.1016/j.ejpb.2015.04.032.

[8] Shikamura Y, Yamazaki Y, Matsunaga T, Sato T, Ohtori A, Tojo K. Hydrogel Ring for Topical Drug Delivery to the Ocular Posterior Segment. Curr Eye Res 2016;41:653–61. https://doi.org/10.3109/02713683.2015.1050738.

[9] Chiang P-C, Hu Y. Simultaneous Determination of LogD, LogP, and pKa of Drugs by Using a Reverse Phase HPLC Coupled with a 96-Well Plate Auto Injector. Comb Chem High Throughput Screen 2009;12:250–7. https://doi.org/10.2174/138620709787581693.

[10] Pereira-da-Mota AF, Vivero-Lopez M, Serramito M, Diaz-Gomez L, Serro AP, Carracedo G, et al. Contact lenses for pravastatin delivery to eye segments: Design and in vitro-in vivo correlations. J Control Release 2022;348:431–43. https://doi.org/10.1016/j.jconrel.2022.06.001.

[11] Burger C, Gerber M, Du Preez JL, Du Plessis J. Optimised transdermal delivery of pravastatin. Int J Pharm 2015;496:518–25. https://doi.org/10.1016/j.ijpharm.2015.10.034.

[12] Ramsay E, Ruponen M, Picardat T, Tengvall U, Tuomainen M, Auriola S, et al. Impact of Chemical Structure on Conjunctival Drug Permeability: Adopting Porcine Conjunctiva and Cassette Dosing for Construction of In Silico Model. J Pharm Sci 2017;106:2463–71. https://doi.org/10.1016/j.xphs.2017.04.061.

[13] Kim HM, Han H, Hong HK, Park JH, Park KH, Kim H, et al. Permeability of the retina and rpe-choroid-sclera to three ophthalmic drugs and the associated factors. Pharmaceutics 2021;13:1–11. https://doi.org/10.3390/pharmaceutics13050655.

[14] Hutton-Smith LA, Gaffney EA, Byrne HM, Maini PK, Gadkar K, Mazer NA. Ocular Pharmacokinetics of Therapeutic Antibodies Given by Intravitreal Injection: Estimation of Retinal Permeabilities Using a 3-Compartment Semi-Mechanistic Model. Mol Pharm 2017;14:2690–6. https://doi.org/10.1021/acs.molpharmaceut.7b00164.

[15] Missel P, Chastain J, Mitra A, Kompella U, Kansara V, Duvvuri S, et al. In vitro transport and partitioning of AL-4940, active metabolite of angiostatic agent anecortave acetate, in ocular tissues of the posterior segment. J Ocul Pharmacol Ther 2010;26:137–45. https://doi.org/10.1089/jop.2009.0132.
